# Meaningful engagement of people living with HIV who use drugs: methodology for the design of a Peer Research Associate (PRA) hiring model

**DOI:** 10.1186/s12954-016-0116-z

**Published:** 2016-10-07

**Authors:** K. Closson, R. McNeil, P. McDougall, S. Fernando, A. B. Collins, R. Baltzer Turje, T. Howard, S. Parashar

**Affiliations:** 1 Faculty of Health Sciences, Simon Fraser University, Burnaby, BC Canada; 2 BC Centre for Excellence in HID/AIDS, St. Paul’s Hospital, 608-1081 Burrard Street, Vancouver, BC V6Z 1Y6 Canada; 3 Dr. Peter AIDS Foundation, Vancouver, Canada; 4 Positive living society and the British Columbia Centre for Excellence in HIV/AIDS, Vancouver, Canada

**Keywords:** Community-based participatory research, Employment, HIV/AIDS, Harm reduction, Drug users

## Abstract

**Background:**

Community-based HIV, harm reduction, and addiction research increasingly involve members of affected communities as Peer Research Associates (PRAs)—individuals with common experiences to the participant population (e.g. people who use drugs, people living with HIV [PLHIV]). However, there is a paucity of literature detailing the operationalization of PRA hiring and thus limited understanding regarding how affected communities can be meaningfully involved through low-barrier engagement in paid positions within community-based participatory research (CBPR) projects. We aim to address this gap by describing a low-threshold PRA hiring process.

**Results:**

In 2012, the BC Centre for Excellence in HIV/AIDS and the Dr. Peter AIDS Foundation collaborated to develop a mixed-method CBPR project evaluating the effectiveness of the Dr. Peter Centre (DPC)—an integrative HIV care facility in Vancouver, Canada. A primary objective of the study was to assess the impact of DPC services among clients who have a history of illicit drug use. In keeping with CBPR principles, affected populations, community-based organizations, and key stakeholders guided the development and dissemination of a low-barrier PRA hiring process to meaningfully engage affected communities (e.g. PLHIV who have a history of illicit drug use) in all aspects of the research project.

The hiring model was implemented in a number of stages, including (1) the establishment of a hiring team; (2) the development and dissemination of the job posting; (3) interviewing applicants; and (4) the selection of participants. The hiring model presented in this paper demonstrates the benefits of hiring vulnerable PLHIV who use drugs as PRAs in community-based research.

**Conclusions:**

The provision of low-barrier access to meaningful research employment described herein attempts to engage affected communities beyond tokenistic involvement in research. Our hiring model was successful at engaging five PRAs over a 2-year period and fostered opportunities for future paid employment or volunteer opportunities through ongoing collaboration between PRAs and a diverse range of stakeholders working in HIV/AIDS and addictions. Additionally, this model has the potential to be used across a range of studies and community-based settings interested in meaningfully engaging communities in all stages of the research process.

**Electronic supplementary material:**

The online version of this article (doi:10.1186/s12954-016-0116-z) contains supplementary material, which is available to authorized users.

## Background

Since the outset of the HIV epidemic, people who use drugs (PWUD) have advocated for more meaningful and egalitarian participation in the processes affecting their lives [1]. This has been done in order to ensure that political and research agendas are relevant, actionable, and genuinely improve the health and well-being of PWUD who are living with, or at risk of, HIV. The ‘nothing about us without us’ approach, which demands the involvement of PWUD in all aspects of HIV policy and program development, has been formally recognized as best practice by national and international agencies, including the International HIV/AIDS Alliance, Open Society Institute, and Canadian HIV/AIDS Legal Network [2, 3]. Similarly, the urgency to work in partnership with people living with HIV (PLHIV) coalesced into a movement recognizing the need for greater and meaningful involvement of PLHIV (GIPA/MIPA) in the global response to the HIV epidemic [1].

The GIPA/MIPA and ‘nothing about us without us’ movements have coincided with, and contributed to, the emergence of community-based participatory research (CBPR) [2]. Within the broader goals of strengthening community capacity and improving quality of life, CBPR aims to generate knowledge about health priorities by empowering and placing affected communities, as well as the community-based organizations (CBOs) that serve them, at the centre of research [4–6]. Increasingly, networks and coalitions of PLHIV, PWUD, CBOs, and international organizations are demanding improved efforts to include affected populations in all stages of the research process, from research grant development to knowledge translation [7, 8].

In response to these appeals, community-based HIV and harm reduction research has increasingly involved members of affected communities as Peer Research Assistants or Associates (PRAs)—members of the affected community with common experiences to the participant population (e.g. experience of homelessness, drug use) who are trained in research activities [9–14]. The involvement of PRAs within CBPR seeks to address existing and historical power imbalances between researchers and participants [15–17]. PRAs often facilitate data collection with the expectation that shared lived experience will increase the disclosure and validity of participants’ self-reported information [18]. Additionally, involving PRAs has been shown to improve community trust, increasing the recruitment of harder-to-reach populations, such as PWUD, while simultaneously providing peer-support opportunities for both PRAs and study participants [6, 16, 17]. Given that PLHIV are disproportionately impacted by social-structural inequities such as homelessness and poverty [19, 20], paid positions are instrumental in ensuring their meaningful participation in research. Recently recognized as a successful form of harm reduction for PWUD, meaningful employment can also be a structural intervention for this population linked to wider improvements in health and social outcomes [21, 22].

While numerous studies focusing on HIV and harm reduction have been undertaken using a CBPR approach [5, 6, 9, 14, 16, 23], there is a paucity of literature detailing the operationalization of PRA hiring. As such, there remain substantial logistical, ethical, and methodological gaps and challenges for researchers who wish to involve PRAs in research [9]. We address these gaps by describing the PRA hiring process for the Dr. Peter Centre (DPC) study.

## Methods

### Development of CBPR partnership

The DPC is an integrative care facility for PLHIV that provides comprehensive HIV and ancillary services, including access to harm reduction services (e.g. supervised injection room, injecting equipment), meal programming, medical care, counselling, and recreation programming [24]. In 2012, the BC Centre for Excellence in HIV/AIDS (BCCfE) and DPC partnered to conduct a mixed-method evaluation of the effectiveness of the DPC’s integrative model of care provided to priority populations of PLHIV in Vancouver [25]. The study objectives specifically examined the impact of the DPC on the health outcomes and quality of life of PLHIV who have a history of illicit drug use. Grounded in a CBPR framework, all aspects of the study design, including development of study instruments and data analysis, were conducted in collaboration with the BCCfE, DPC staff, key community stakeholders, and peers (i.e. people living with HIV, including clients of the Dr. Peter Centre). As part of the development of the DPC study, focus groups were held in order to inform the research team of DPC client priorities. Throughout the focus groups, participants stressed the importance of the ‘nothing about us without us’ principles, emphasizing the need for increased meaningful involvement in research and highlighting gaps in opportunities for paid positions within research.

Subsequent to the focus group discussions, a Community Advisory Committee (CAC) comprised of DPC clients and other community stakeholders (e.g. funders, policymakers) was developed to guide the investigative team to ensure the study was grounded in the priorities of the community. Furthermore, to address community-identified gaps in paid employment opportunities within research, the CAC and investigative team developed and implemented a hiring process that aimed to engage key populations of PLHIV, including those who previously or currently use illicit drugs, as PRAs within the DPC study.

## Results

### PRA hiring model

For the purposes of the DPC study, PRAs were defined as PLHIV who had experiences and identities in common with the study participants (e.g. histories of mental health challenges, homelessness, illicit drug use), and included non-DPC clients. The CAC and investigative committee collaboratively decided upon this definition in consultation with other CBPR studies with the aim of hiring PRAs whose lived experience reflected the experiences of prospective study participants.

While PRA hiring is typically conducted solely by the research institution, our approach involved members of the DPC staff taking responsibility for hiring and supervising the PRAs in coordination with the investigative team. The hiring process adhered to the existing organizational hiring policies and procedures of the Dr. Peter AIDS Foundation (DPAF), the non-profit organization that raises funds to operate the DPC. In addition, the hiring team consulted job postings from other relevant research projects and a working paper outlining potential recruitment and selection approaches when hiring PRAs [26–28]. We aimed to develop and implement a low-barrier hiring process, ensuring the equitable representation and engagement of communities of PLHIV who use illicit drugs that have been previously excluded from paid positions within research initiatives [29].

### Establishment and composition of the hiring team

**Fig. 1:**
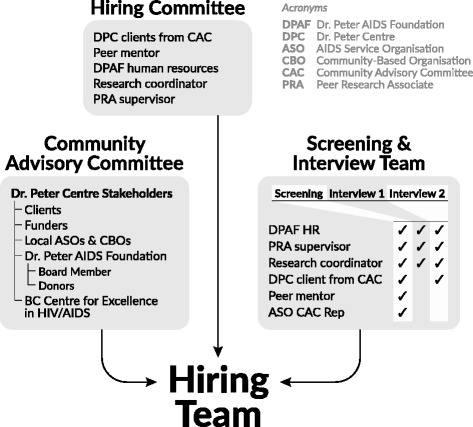
Peer Research Associate hiring organization chart

The hiring team, comprised of three sub-groups with different functions, was established to execute the hiring process as follows: (i) the hiring committee guided the overall hiring process and included a Human Resources representative and the PRA supervisor from the DPAF, as well as the research coordinator, a PRA mentor with significant experience in CBPR, and DPC clients from the CAC; (ii) a screening team screened the applications and included members of the hiring committee as well as a representative from a local AIDS service organization (ASO) who was on the CAC; (iii) and finally, interview teams conducted the interviews (see Fig. 1). All members of the CAC were invited to join the hiring team; however, to maximize inclusivity, CAC members who were interested in applying for the position were asked not to join. Ultimately, only one CAC member declined to join the hiring team due to an intention to apply for the position. Other CAC members declined to join the hiring team due to other time commitments. Engaging with a variety of stakeholders on the hiring team introduced different perspectives and ensured community involvement in the process.

### Development and dissemination of the job posting

**Fig. 2:**
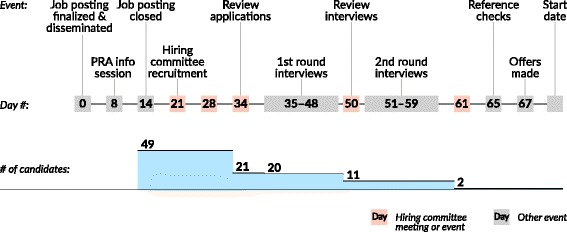
Peer Research Associate hiring schedule

A detailed description of the PRA hiring schedule is outlined in Fig. 2 and began with the development of a one-page plain language job description and application form, written at a grade 8 reading level to ensure accessibility in the recruitment process (see Additional file 1). The job description outlined the roles and responsibilities of the position and listed specific competencies (e.g. able to work as part of a diverse team, basic verbal communication skills). The application form consisted of a single question: ‘Please tell us why you are interested in working as a PRA on this project. Feel free to list your interests or any previous research related experience. You can also submit a resume, but this is not required.’ This question was designed to place value on prospective applicants’ wide range of previous work and volunteer experiences, prioritize the lived experiences of the DPC clientele, and in line with international guidelines for the meaningful employment of PWUD, offer an opportunity to those with limited or no formal experience to apply on the basis of their interest in the position [3].

Two PRA positions were advertised as 12-month term positions, with the intent of providing opportunities to more than two people over the course of the 3-year project. The job was posted at the DPC and disseminated through community networks and allied organizations (e.g. ASOs, drug user-led organizations).

An information session was hosted by the study research coordinator, PRA mentor, and PRA supervisor to answer prospective applicants’ questions regarding the PRA position. The location and time of the information session were listed in the job posting, and 25 individuals attended. The information session described the study’s community-based approach and objectives, as well as PRA job duties and responsibilities. This was followed by a 30-min question and answer session. Questions from attendees included compensation details, whether abstaining from particular drugs was required to be eligible, and logistical questions (e.g. start date, hours of work). Attendees were assured that drug abstinence was not a requirement for the position and were informed that they would not be asked about current drug use.

### Interviewing applicants

In 2013, the DPC received 49 applications to fill the two PRA positions. The screening team reviewed the initial applications using criteria that referred to competencies defined in the job description (see Additional file 1). From the 49 applications, 21 applicants met core competencies (i.e. showed evidence in their application of having worked as part of a diverse team, or previous experience completing similar tasks as described in the posting) and were thus selected for a round 1 interview. Interview questions were developed with the input of the hiring committee and the study’s investigative team, and refined by the DPAF Human Resources representative. This collaborative process was necessary to ensure that all questions were in accordance with DPAF Human Resources policies, procedures, and employment standards.

The DPAF Human Resources representative, PRA supervisor, and the study coordinator interviewed 20 applicants (one candidate withdrew), with each interview lasting 15 min. Applicants were asked to describe themselves and previous experiences that made them a good fit for the position, as well as discuss a time when they were part of a team or diverse group working towards a common goal. The round 1 interview team used the application screening criteria to evaluate candidates during these interviews. The purpose of the round 1 interview was to narrow down the list of candidates using the same criteria as was used in the application screening process. At every stage of the 2013 and 2014 hiring processes, candidates were selected for the next stage according to specific criteria that reflected the competencies listed in the job description (see Additional file 1). In 2013, 11 candidates were selected for a second interview.

The second round interviews were conducted by a DPAF Human Resources representative, the PRA supervisor, the study coordinator, and a DPC client representative from the CAC. Interviews lasted approximately 45 min, and all clients were assessed using an interview guide (see Additional file 2). To measure the performance of each candidate during the first and second round interviews, each candidate was scored following a letter grade schema: A+, A, A−, B+, B, B−, C+, C, C−, D. At the end of all the interviews, the letter grades were translated into a number in order to develop a means to compare the candidates: D = 1, C− = 2, C = 3 […] A+ = 10. A candidate’s score for each category was summed up into a total score. Candidates were ranked according to their total score, and the selection for the round 2 interview and selection for the position were based on those scores. In the first round, certain criteria could not be ascertained through the 15-min interview (reading skills, and basic computer skills). These criteria were designated as ‘not applicable.’ As an asset qualification, candidates were not penalized if they did not have previous research experience (i.e. any mention of previous research experience was noted but not graded, thus this criterion did not impact the candidate’s score). Following the second round of interviews in 2013, two candidates were selected (see Fig. 2).

In 2014, The DPC received 17 applications for two PRA positions. The decreased number of applicants in the second year is thought to be due to concurrent CBPR projects in the community, which resulted in the creation of alternative opportunities to which people could apply. Additionally, interacting with PRAs during and outside the survey administration process helped prospective applicants better understand the job requirements, which may have influenced recruitment. Ten candidates were selected for an interview. Due to the smaller numbers, it was deemed unnecessary to hold a second round of interviews.

Candidates who were not selected were contacted and informed at each stage in the hiring process. This unique addition to the existing DPAF hiring process was deemed necessary by the hiring committee to respectfully thank applicants for applying, rather than simply not informing unsuccessful candidates, as can be the norm in some hiring processes. This hiring process was piloted during the first year of the study and revised to promote continuous improvement in the second year of the study. In total, five PRAs were hired over a span of 2 years.

## Discussion

This PRA hiring process demonstrates how community-based organizations and researchers can operationalize ‘nothing about us without us’ and GIPA principles in the context of a CBPR partnership to promote the involvement of affected populations in research, including people who use illicit drugs. By harnessing different strengths of key community stakeholders and affected populations, this process sought to create a consistent, low-barrier, and equitable recruitment and hiring process. Five PRAs were successfully hired as DPC employees over a 2-year period using the process outlined above to assist in the planning and execution of the DPC study. Additionally, this hiring process contributed to a unique and innovative employment opportunity and data collection strategy that should be considered within future CBPR projects.

Receiving 49 and 17 applications during the first and second years of the DPC study, respectively, suggests a strong interest in peer-based vocational opportunities among the affected community. Importantly, the affected community for this study—PLHIV with histories of illicit drug use, mental illness, and unstable housing—faces multiple social-structural barriers to engaging in formal employment, particularly those who have been out of the workforce for long periods of time [30]. The 12-month position aimed to allow for numerous opportunities for affected community members to become involved in community-based research projects. Additionally, the position provided an opportunity for interested applicants to re-apply in subsequent years if they were not recruited initially.

The hiring of PRAs within our study provides a potential avenue to address structural inequities to equal employment opportunities for PLHIV with a history of illicit drug use in a Canadian context. PRAs gained research-based skills over their contracted time, which were transferable into more permanent semi-professional positions (e.g. peer mentor, peer navigator) following the end of their term. This is significant as high levels of workplace stigma and discrimination create numerous barriers for PLHIV and PWUD wishing to enter into the workplace and maintain formal employment [31–33]. This lack of opportunities often results in increased engagement in informal, street-based income generation (e.g. selling drugs, street-based sex work), which has been associated with heightened experiences of violence [30] and a decreased likelihood of achieving drug cessation [28]. Low-barrier formal employment opportunities have previously been associated with improved ART adherence and life expectancy for high-risk affected populations of PLHIV [33, 34]. Thus, our hiring process aimed to provide an avenue for addressing the deleterious effects of employment disparities for marginalized populations including PLHIV and PWUD [3].

The development and dissemination of this hiring model adds to the literature on best practices for employing PWUD and has a number of implications for future CBPR initiatives [3]. Traditionally, there has been a lack of monetary compensation for PRAs’ participation within research projects [3, 23, 28]. In line with ‘nothing about us without us’ principles, we acknowledge that PWUD and PLHIV provide invaluable lived expertise to the research process and thus should be equally and equitability compensated for their contributions. This hiring process was largely delegated to the CBOs in order to overcome gaps in methods used to include PRAs in the research process. From the development of the job description to the selection of successful applicants, the DPC was at the forefront of the process, and DPC clients were meaningfully involved. Ensuring that DPC clients were represented throughout the hiring process demonstrated that the perspectives of PLHIV were strongly valued. In this regard, championing GIPA principles within hiring processes helps to address concerns around tokenistic involvement of PLHIV and PWUD [13, 23, 30], and clearly establishes tools for meaningful engagement.

This hiring process has been adapted to meet the needs of individual community-academic collaborations wishing to involve PRAs in their research projects [35]. There are a number of factors that future CBPR initiatives may wish to consider when engaging affected populations as key members of the research team, such as time and resource commitments. To reduce any biases stemming from prior interaction between hiring committee and applicants, future CBPR-based hiring processes may want to blind initial screening. It is important to have a strong understanding of the motives for conducting research informed by lived experiences, and how the inclusion of PRAs fits within the visions and values of studies.

Hiring guidelines and payment procedures within CBPR projects that include PRAs as paid employees must be transparent. For example, CBOs need to carefully consider how to define PRA roles—both as employees and clients—within their organizations, including the type of work (e.g. part-time, casual, volunteer) and the nature of remuneration (e.g. salary, hourly wage, honorarium). Furthermore, community and academic partners should align their hiring process with the existing Human Resources policies of the organization where the successful applicants will be working.

This paper has several limitations that should be noted. Although the hiring process sought to create low-barrier, equitable opportunities for PLHIV who use drugs, further research is needed to evaluate the impact of PRA hiring processes and the use of ‘nothing about us without us’ principles on the quality of data collection as well as participant and PRA experiences. Future research could include more rigorous process evaluations involving various types of data collection (e.g. focus groups, ethnographic observation, interviews) with all stakeholders involved in the hiring process. Such evaluations should also consider the impact of peer-informed hiring models on the self-perception and sense of ownership of PRAs within CBPR projects. Finally, while vocational opportunities for PLHIV may produce long-term benefits, we were unable to determine the nature and extent of these benefits.

## Conclusion

The hiring process described here aimed to ensure that the DPC study was conducted in the interest of the community and that PLHIV who use drugs were acknowledged for their expertise acquired through lived experience. Furthermore, this model and community-based study were developed to ensure that peers were empowered to build research facilitation capacity and share research results. Future CBPR studies aiming to hire PRAs should consider using similar guidelines to ensure applicants are treated equitably, and also widely disseminate details of their hiring processes to add to the literature on engaging community members and affected populations in research. In this way, the research findings and methods contribute to positive, relevant, and actionable change that improves the health and well-being of affected communities.

## Additional files

Additional file 1: Job application form (DOCX 130 kb)
Additional file 2: Interview guide (DOCX 127 kb)

## Abbreviations

ASO: AIDS service organization
BCCfE: BC Centre for Excellence in HIV/AIDS
CAC: Community Advisory Committee
CBOs: Community-based organizations
CBPR: Community-based participatory research
DPAF: Dr. Peter AIDS Foundation
DPC: Dr. Peter Centre
GIPA/MIPA: Greater and meaningful involvement of people living with HIV/AIDS
PLHIV: People living with HIV
PRA: Peer Research Associate
PWUD: People who use drugs

## References

[1] The Joint United Nations Programme on HIV/AIDS (UNAIDS): From principle to practice: greater involvement of people living with or affected by HIV/AIDS (GIPA). Geneva, Switzerland 1999.

[2] Canadian HIV/AIDS Legal Network: "Nothing about us without us" greater, meaningful involvement of people who use illegal drugs: a public health, ethical, and human rights imperative Vancouver, BC 2006.

[3] International HIV/AIDS Alliance: Good practice guide for employing people who use drugs United Kingdom 2015.

[4] Minkler M, Blackwell AG, Thompson M, Tamir H (2003). Community-based participatory research: implications for public health funding. Am J Public Health.

[5] Wallerstein NB, Duran B (2006). Using community-based participatory research to address health disparities. Health Promot Pract.

[6] Wu L, Li X (2013). Community-based HIV/AIDS interventions to promote psychosocial well-being among people living with HIV/AIDS: a literature review. Health Psychol Behav Med.

[7] Ontario AIDS Network: Living and serving 3: GIPA engagement guide and framework for Ontario ASOs. Ontario AIDS Network 2011

[8] UNAIDS: Peer education and HIV/AIDS: Concepts, uses and challenges. Geneva, Switzerland 1999

[9] Lazarus L, Shaw A, LeBlanc S, Martin A, Marshall Z, Weersink K, Lin D, Mandryk K, Tyndall MW, Committee PCA (2014). Establishing a community-based participatory research partnership among people who use drugs in Ottawa: the PROUD cohort study. Harm Reduct J.

[10] Carter A, Greene S, Nicholson V, O'Brien N, Sanchez M, de Pokomandy A, Loutfy M, Kaida A, Canadian HIVWsS, Reproductive Health Cohort Study Research T (2015). Breaking the glass ceiling: increasing the meaningful involvement of women living with HIV/AIDS (MIWA) in the design and delivery of HIV/AIDS services. Health Care Women Int.

[11] Flicker S, O'Campo P, Monchalin R, Thistle J, Worthington C, Masching R, Guta A, Pooyak S, Whitebird W, Thomas C (2015). Research done in "a good way": the importance of indigenous elder involvement in HIV community-based research. Am J Public Health.

[12] Greene S, Tucker R, Rourke SB, Monette L, Koornstra J, Sobota M, Byers S, Hwang S, Dunn J, Guenter D (2010). "Under my umbrella": the housing experiences of HIV positive parents who live with and care for their children in Ontario. Arch Womens Ment Health.

[13] Guta A, Flicker S, Roche B (2013). Governing through community allegiance: a qualitative examination of peer research in community-based participatory research. Crit Public Health.

[14] Guta A, Strike C, Flicker S, Murray SJ, Upshur R, Myers T (2014). Governing through community-based research: lessons from the Canadian HIV research sector. Soc Sci Med.

[15] Brizay U, Golob L, Globerman J, Gogolishvili D, Bird M, Rios-Ellis B, Rourke SB, Heidari S (2015). Community-academic partnerships in HIV-related research: a systematic literature review of theory and practice. J Int AIDS Soc.

[16] Greene S (2013). Peer research assistantships and the ethics of reciprocity in community-based research. J Empir Res Hum Res Ethics.

[17] Jagosh J, Macaulay AC, Pluye P, Salsberg J, Bush PL, Henderson J, Sirett E, Wong G, Cargo M, Herbert CP (2012). Uncovering the benefits of participatory research: implications of a realist review for health research and practice. Milbank Q.

[18] Elliott E, Watson AJ, Harries U (2002). Harnessing expertise: involving peer interviewers in qualitative research with hard-to-reach populations. Health Expect.

[19] Milloy MJ, Kerr T, Bangsberg DR, Buxton J, Parashar S, Guillemi S, Montaner J, Wood E (2012). Homelessness as a structural barrier to effective antiretroviral therapy among HIV-seropositive illicit drug users in a Canadian setting. AIDS Patient Care STDS.

[20] Rourke SB, Sobata M, Tucker R, Bekele T, Gibson K, Greene S, Positive Spaces HPT (2011). Social determinants of health associated with hepatitis C co-infection among people living with HIV: results from the positive spaces, healthy places study. Open Med.

[21] Richardson L, Wood E, Kerr T (2013). The impact of social, structural and physical environmental factors on transitions into employment among people who inject drugs. Soc Sci Med.

[22] Richardson L, Sherman SG, Kerr T (2012). Employment amongst people who use drugs: a new arena for research and intervention?. Int J Drug Policy.

[23] Logie C, James L, Tharao W, Loutfy MR (2012). Opportunities, ethical challenges, and lessons learned from working with peer research assistants in a multi-method HIV community-based research study in Ontario, Canada. J Empir Res Hum Res Ethics.

[24] Griffiths H (2002). Dr. Peter Centre—removing barriers to health care services. Nurs BC.

[25] "Dr. Peter AIDS Foundation ". Retrieved April 30th, 2016, from. http://www.drpeter.org/dr-peter-centre/.

[26] Norman T, Pauly B, Greater Victoria Coalition to end homelessness: engendering dialogue and meaningful participation among constituencies working toward ending homelessness in Victoria, BC. British Columbia; Ontario AIDS Network, University of Victoria

[27] Canadian HIV Women's Sexual and Reproductive Health Cohort Study (CHIWOS) PRA Working Group: CHIWOS Peer Research Associate Training Manual: English Quebec Version. Toronto 2013

[28] Roche B, Guta A, Flicker S: Peer research in action I: models of practice. Toronto; The Wellesley Institute 2010

[29] Coupland H, Maher L (2005). Clients or colleagues? Reflections on the process of participatory action research with young injecting drug users. Int J Drug Policy.

[30] Richardson L, Small W, Kerr T (2016). Pathways linking drug use and labour market trajectories: the role of catastrophic events. Sociol Health Illn.

[31] UK Drug Policy Commission: getting problem drug users (back) into employment: part two London; 2008

[32] Ti L, Richardson L, DeBeck K, Nguyen P, Montaner J, Wood E, Kerr T (2014). The impact of engagement in street-based income generation activities on stimulant drug use cessation among people who inject drugs. Drug Alcohol Depend.

[33] Nachega JB, Uthman OA, Peltzer K, Richardson LA, Mills EJ, Amekudzi K, Ouedraogo A (2015). Association between antiretroviral therapy adherence and employment status: systematic review and meta-analysis. Bull World Health Organ.

[34] Richardson L, DeBeck K, Feng C, Kerr T, Wood E (2014). Employment and risk of injection drug use initiation among street involved youth in Canadian setting. Prev Med.

[35] Cain R, Collins E, Bereket T, George C, Jackson R, Li A, Prentice T, Travers R. Challenges to the involvement of people living with HIV in community-based HIV/AIDS organizations in Ontario, Canada. AIDS Care. 2014;26:263–6. 10.1080/09540121.2013.803015 23724932

