# Supplementary material for: Meaningful engagement of people living with HIV who use drugs: methodology for the design of a Peer Research Associate (PRA) hiring model
Source: Harm Reduct J. 2016 Oct 7;13:26. doi: 10.1186/s12954-016-0116-z (PMC5054577; doi:10.1186/s12954-016-0116-z)
Supplement: Additional file 1: — Job application form (DOCX 130 kb) [file 12954_2016_116_MOESM1_ESM.docx]

Additional file 1: Job application form

**Job Posting: Peer Research Associate (2 Positions Available)**

**Dr. Peter AIDS Foundation**

**Introduction:**

The Dr. Peter AIDS Foundation and the BC Centre for Excellence in HIV/AIDS have partnered with researchers from across Canada to do a three-year research study on the Dr. Peter Centre (DPC). This study is funded by the Canadian Institutes of Health Research and the Michael Smith Foundation for Health Research. One part of the study is to do a survey with DPC clients. We are currently seeking two Peer Research Associates (PRAs) to administer this computer-based survey at the DPC.

**About the Job:**

- As part of the job, you will help to recruit DPC clients for the survey and administer the survey to DPC clients. To administer the survey, you will need to relay information about the survey, explain survey instructions and enter survey responses on the laptop.
- The position will also involve a number of tasks such as: obtaining consent from DPC clients doing the survey, administering the honorarium to DPC clients who participate in the survey, maintaining records and completing paperwork, and managing the laptop when the survey is being done. You will also work collaboratively with the other PRA.
- We are looking for people who are passionate about doing this job, able to work as part of a diverse team and who have reading, comprehension, verbal communication and basic computer skills. You are not required to have previous research experience, but previous experience is an asset. The expectation is that you would be a fit with the description of a PRA for this job (please see below: “About PRAs for this Job”).
- This is a part time 12-month term position for up to 18 hours a week with an hourly rate of $16.32. The intent is to provide opportunities to more than two people over the course of the three-year project (so, if you don’t get the job for this round, you can apply again for the next round in a year).

**About PRAs for this Job:**

For this job, PRAs are defined as persons living with HIV/AIDS who have experiences and identities in common with people who will do the survey (DPC clients). Both DPC clients and non-DPC clients can apply for these positions.

**About the Dr. Peter Centre:**

The Dr. Peter Centre is an HIV/AIDS health care facility located in Vancouver’s West End. The Centre provides support to people living with HIV/AIDS who also face issues such as poverty, homelessness, mental health and addiction.

**Information Session about the Job:**

There will be an information session about the job on Wednesday June 12 at 4pm at the Dr. Peter Centre (1110 Comox Street, Vancouver).

**How to Apply:** Please follow the instructions on the application form on the next page. Please note the deadline for applications is: **Tuesday June 18, 2013 at 4pm.**

**Peer Research Associate (PRA) Application Form**

Application Instructions: Please complete and submit this application form via email, fax or in-person to the Dr. Peter Centre (details below). Please make sure that you have fully completed the application form and your responses are written clearly.

**Application deadline is: Tuesday June 18, 2013 at 4pm**

| First Name: | | Last Name: | |
| --- | --- | --- | --- |
| Address: | City: | | Postal Code: |
| Phone: | Other Means of Contact: | | Best way to contact you: |
| Email: | | | |
| Please tell us why you are interested in working as a PRA on this project. Feel free to list your interests or any previous research related experience. You can also submit a resume, but this is not required. | | | |

**Drop-off applications at Reception at the Dr. Peter Centre or send applications via fax or email:** Dr. Peter Centre, 1110 Comox Street (Vancouver) • Fax: 604-608-4259 • Email: PRA@drpeter.org
