# Supplementary material for: Meaningful engagement of people living with HIV who use drugs: methodology for the design of a Peer Research Associate (PRA) hiring model
Source: Harm Reduct J. 2016 Oct 7;13:26. doi: 10.1186/s12954-016-0116-z (PMC5054577; doi:10.1186/s12954-016-0116-z)
Supplement: Additional file 2: — Interview guide (DOCX 127 kb) [file 12954_2016_116_MOESM2_ESM.docx]

**Additional file 2: Interview guide**

**Interview Guide for the PRA Position**

[DPAF Supervisor]: Hello and thank you very much for meeting with us today. We will be using both a script & a timer to ensure that the interview process is fair and equal for everyone. This interview will be 40 minutes in length. You will be asked 12 questions. All individuals will be asked the same 12 questions, and the last question will be “Do you have any questions for us?”:

1. [DPC Client]: **Briefly**, what do you know about the Dr. Peter Centre?
2. [DCPS Coordinator]: Many residents/participants at the Dr. Peter Centre have histories of mental illness, drug addiction, and extreme poverty. They have diverse backgrounds: gay, transgendered, first nations, street involved, prison histories etc. Do you have experience working with any of these populations? If so, can you tell us about your experience(s) in doing so?
3. [DPAF Supervisor]: For this next question I am handing to you a list of key PRA tasks to look at.

Being a PRA is demanding work. As a PRA on this project, you will need to obtain informed consent (through standard consenting procedures) and administer the same survey over and over again to participants. This process will involve explaining instructions, communicating questions clearly, and dealing with information of a sensitive nature for people living with various life challenges. You will be responsible for ensuring the appropriate lock-up and security of study-related materials that you handle (i.e. laptop, swipe cards, information, etc.). You will also be attending regular meetings, check-ins and, if you are interested in doing so, you may also be called upon to assist with other aspects of the study at various times during the course of the project (i.e. such as KT dissemination – help in presenting findings to the community/public, feedback on the process, etc.). All job-related responsibilities will take place within work hours. You will not be required to perform any work related tasks outside of these hours. Over the course of the 1-year term, you may have the opportunity to attend conferences, and other study-related events.

Given this information, what do you think you will find challenging about this position? How might you handle these challenges? What might you find rewarding about this position?

1. [DPC Client]: Each PRA would be doing these tasks independently, but there is also a lot of work that is done together. Please describe a time you worked well in a team or partnership.
2. [DPC HR representative]: When administering a survey, you may come up against a participant who is challenging in some way (for example: falling asleep or behaving in a distracted was, very obviously rushing through questions just to get the honorarium, becoming agitated and saying you have no right to ask personal questions, crying and oversharing stories that are not relevant to the interview). If you were administering the survey to a participant dealing with one or some of these issues, how would you respond? How do you bring the participant back on the present and back on task?
3. [DPCS Coordinator]: This next question allows us to see your comfort level with computers. Here is a computer with a sample of the survey that will be used in this study [explain basic functionality of the survey]. You are the PRA, and I am the participant. I would like you to take me through the next three screens of questions.
4. [DPC Client]: Previous research experience is not required for this position, but if you happen to have any previous research experience, or skills you have gained from other experiences that you think would apply/be relevant to this position, can you share what those are with us? This could include having completed surveys as an interviewee.
5. [DPC Supervisor]: The Dr. Peter Centre Day Health program is open from 9am – 3pm, and we would be doing the surveys during this time. Can you tell me about your availability over the course of a week? Training for the position will likely be for 2 full days in November, do you see any issues around your availability for the training?
6. [DPC HR representative]: The hours and the wage of the position are as follows: up to 15 hours a week with an hourly rate of $16.32. Is there anything with this level of earnings that will cause an issue for you?
7. [DPC Coordinator]: If required, would you be able to provide me with the name and phone number of 2 to 3 references?
8. [DPC HR coordinator]: As part of the hiring process, all new staff members of the Dr. Peter AIDS Foundation are required to undergo a criminal record check. The criminal record check will only screen for things that might come in conflict with the work that we do with vulnerable populations. In addition, it will only be conducted in the event that you are considered for the position. If you are comfortable allowing us to do a criminal record check, we will need you to sign a consent form.
9. [DPC Client]: Do you have any questions for us?

[DPC Supervisor]: Thank you very much for meeting with us today. This concludes the interview. You can expect to hear back from us within the next two weeks (or sooner). All individuals will be contacted and notified of their application status. Once again, thank you very much.
